# Past successes and future opportunities for the genetics of the human Y chromosome

**DOI:** 10.1007/s00439-017-1806-7

**Published:** 2017-04-29

**Authors:** Yali Xue, Chris Tyler-Smith

**Affiliations:** The Wellcome Trust Sanger Institute, Wellcome Genome Campus, Hinxton, Cambs, CB10 1SA UK

**Fig. 1:**
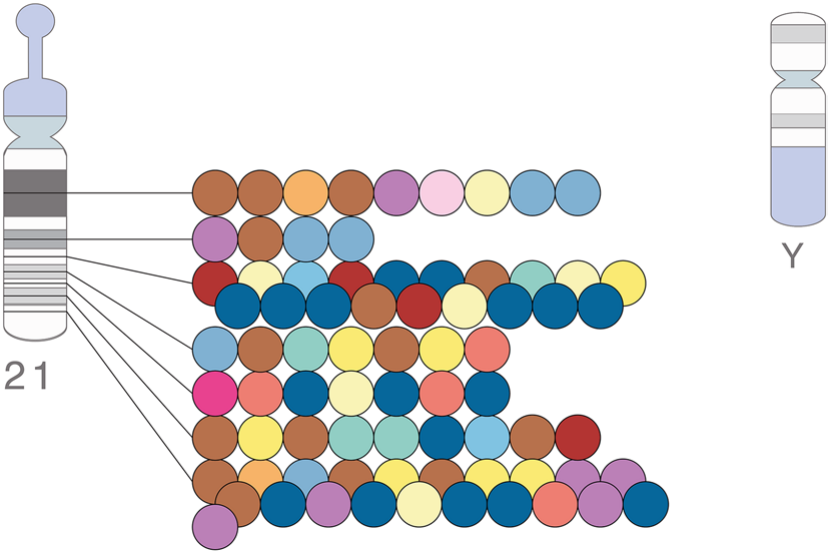
Lack of GWAS hits on the human Y chromosome, compared with chromosome 21. Each *circle* represents a GWAS hit, with the *color* indicating the class of phenotype. See https://www.ebi.ac.uk/gwas/diagram for further details

The Y chromosome has a special primary role in human genetics—determining male sex—that gives it a functional unity in a way that no other chromosome has, and leads to a host of unique consequences for its medical, population and forensic genetics. However, partly as a result of its distinctness from the rest of the genome plus its rather complicated structure, it is grossly neglected by most geneticists. Consider, as one illustration, the number of GWAS hits on the Y chromosome compared to a similar-sized autosome such as chromosome 21: zero versus 75 (https://www.ebi.ac.uk/gwas/diagram, accessed on 24th April 2017; Fig. 1). Does Y chromosome variation really have no influence on any of the traits or disease phenotypes that have been examined, or have the right analyses just not been performed? There are thus good reasons to have a Special Issue of *Human Genetics* focussed on the Y chromosome, following on from ones on genome editing, exome sequencing and other exciting advance in methodology and understanding: to celebrate the insights that have been derived from it, and stimulate further work.

Two original investigations document Y-chromosomal variation in the two largest populations in the world: China via typing Y-STRs in ~38,000 men (Nothnagel et al. 2017) revealing a north–south gradient in the Han together with some genetically distinct ethnicities, and India via full re-sequencing of a small, but carefully chosen, set of 42 Y chromosomes (Mondal et al. 2017) highlighting the unique genetics of the people from the Andaman Islands. The rest of the Special Issue offers probably the most comprehensive collection of review articles ever on the human Y chromosome. An evolutionary context is provided by comparing the human Y with those of the other great apes (Hallast and Jobling 2017), revealing the rapidity of its evolution in structure and gene content. Within the human lineage, Y sequences derived from ancient DNA are beginning to transform our understanding of the real complexity of our past, revealing continuity in regions such as the Americas, but repeated replacements in others such as Eurasia (Kivisild 2017). Analyses of present-day Y chromosomes continue to reveal the differences between male and female history, and sometimes extraordinarily abrupt expansions in male numbers (Batini and Jobling 2017). For time periods within the last few generations, the Y chromosome is the most popular tool for investigating genetic genealogy, with much of the work led by citizen, rather than paid, scientists (Calafell and Larmuseau 2016). Many of these studies depend on an understanding of the mutation rates of both Y-SNPs and Y-STRs, which allow calibration of phylogenies— a topic with long-standing controversies that are perhaps approaching some resolution (Balanovsky 2017). An additional form of variation, that in the number of copies of some segments of Y-DNA, is extensive yet under-studied and has implications for population, medical and forensic genetics (Massaia and Xue 2017). Mutational processes on the Y chromosome are, in some ways, simpler than those on other chromosomes because of the lack of recombination with a homolog, but the presence of extensive regions that are repeated within the chromosome itself creates abundant opportunities for gene conversion, influencing their evolution and perhaps function (Trombetta and Cruciani 2017). The Y chromosome has had an important role in forensic genetics for the last 25 years, where STRs are the variants of choice, and difficult cases may take up to 14 years to solve (Kayser 2017). In medical genetics, the Y chromosome’s relevance to spermatogenesis and male fertility has also been apparent for decades, and the importance of variation in copy number of some key genes is well-established, but clinical questions remain (Krausz and Casamonti 2017), while the influence of somatic loss of the chromosome from a proportion of cells is associated with a growing number of conditions of wide medical relevance in elderly men (Forsberg 2017).

Thirty years ago, we knew from cytogenetic studies about the importance of the Y chromosome for sex determination (Jacobs and Strong 1959) and spermatogenesis (Tiepolo and Zuffardi 1976), and that it tended to be lost from the blood cells of elderly men (Jacobs et al. 1963). The first DNA variants on the chromosome had just been reported (Casanova et al. 1985, Lucotte and Ngo 1985). The articles in this Special Issue highlight the extraordinary insights we have gained in different fields since then, especially using next-generation sequencing. However, there are still plenty of unanswered questions on the Y chromosomes, as it is the chromosome with the highest proportion of its DNA inaccessible to short-read sequencing, making up nearly two-thirds of the euchromatin and all of the heterochromatin. Current sequence-based studies of the Y chromosome are restricted to 10 Mb or less of the chromosome, yet much of its medical importance may still hide in the complex regions, which carry most of the spermatogenic genes. Long-read sequencing technologies should soon enable us to access the sequence of the entire chromosome in any individual of interest (Jobling and Tyler-Smith 2017). We can thus look forward to a time in the near future when we will have all the genetic data that can be generated, and can fully explore the roles and uses of the human Y chromosome, perhaps even adding some GWAS hits.
